# Geographical Distances Between Separated Parents: A Longitudinal Analysis

**DOI:** 10.1007/s10680-017-9437-1

**Published:** 2017-07-24

**Authors:** Michael J. Thomas, Clara H. Mulder, Thomas J. Cooke

**Affiliations:** 1 Population Research Centre, Faculty of Spatial Sciences, University of Groningen, P.O. Box 800, 9700 AV Groningen, The Netherlands; 2 Department of Geography, University of Connecticut, 215 Glenbrook Road, U-4148, Storrs, CT USA

**Keywords:** Separation and divorce, Spatial (im)mobility, Family migration, Linked lives, Random-effects models, Great Britain

## Abstract

Using detailed geocoded microdata from the British Household Panel Survey and longitudinal random-effects models, we analyse the determinants and trajectories of geographical distances between separated parents. Findings of particular note include the following: (1) post-separation linked lives, proximities and spatial constraints are characterised by important gender asymmetries; (2) the formation of new post-separation family ties (i.e. new partners and children) by fathers is linked to moves over longer distances away from the ex-partner than for mothers; (3) the distribution of pre-separation childcare responsibilities is relevant for determining post-separation proximity between parents; and (4) most variation in the distance between ex-partners occurs in the immediate period following separation (approximately the first year), suggesting that the initial conditions around separation can have long-lasting implications for the types of family life, ties and contact experienced in the years after separation.

## Introduction

Many Western societies have witnessed long-term trends of increased separation and divorce, increased fathers’ involvement in parenting and the interrelated growth in extended-family complexity. Upon separation, many separated parents will feel a need to remain close to the ex-partner because they want to share parenting responsibilities or facilitate child visitation (Flowerdew and Al-Hamad 2004; Stjernström and Strömgren 2012; Bakker and Mulder 2013). Yet, despite the benefits of improved child–parent access, the coordination and maintenance of geographical proximity will naturally place constraints on parents’ post-separation mobility careers and their ability to find an appropriate residential location. That is, maintaining post-separation proximity could work to restrict residential mobility and migration linked to individual adjustment and recovery processes after separation, including those related to the formation of new family ties.

Given the considerable rise of the post-separation family, an increasingly substantial body of work has emerged documenting the influence of separation on residential mobility and migration (e.g. Courgeau 1985; Flowerdew and Al-Hamad 2004; Feijten and van Ham 2007, 2013; Dewilde 2008, 2009; Mulder and Wagner 2010; Mulder and Malmberg 2011; Clark 2013; Dommermuth 2016; Cooke et al. 2016; Das et al. 2016; Thomas et al. 2017). This literature indeed demonstrates how post-separation mobility is spatially constrained, with several studies showing separated people to move more frequently, but over shorter distances, than the general population (Feijten and van Ham 2007, 2013). Unfortunately, much of this work has drawn on short snapshots of data, offering little potential for the analysis of longer-term mobility careers of separated families.

A wider focus on the significance of linked family lives for spatial (im)mobility behaviour would seem beneficial for population researchers and academics alike. In particular, gender asymmetry in the ability and/or desire of mothers, as compared to fathers, to break with post-separation ties may be an important and currently underappreciated factor behind the various inequalities observed between men and women in the post-separation context (see Bianchi et al. 1999; Uunk 2004; Andreß et al. 2006; Dewilde and Uunk 2008)—constraining mobility that could otherwise help in post-separation adjustment, recovery and well-being. While conceptual work has emphasised how individual (im)mobility decisions and outcomes are formed with reference to the location of significant others (Bailey 2009; Mulder and Cooke 2009; Coulter et al. 2016), its empirical demonstration remains rare. Indeed, given the ubiquitous nature of family instability and complexity across many contemporary Western societies, the accumulation of post-separation linked lives and spatial constraints could be a factor behind corresponding declines in aggregate mobility and migration rates (Cooke 2011, 2013; Bell and Charles-Edwards 2013; Champion and Shuttleworth 2016a, b).

The analysis in this paper seeks to identify the *longer*-*term* determinants and trajectories of post-separation family ties and proximity. Through the application of longitudinal random-effects models on data from the British Household Panel Survey (BHPS), we emphasise the persistence and significance of linked family lives for post-separation (im)mobility behaviour, revealing the critical interactions between the formation of new ties and the maintenance of old ones. More specifically, our analysis demonstrates how post-separation linked lives, proximities and spatial constraints are characterised by important gender asymmetries: the formation of new family ties (i.e. new partners and children) by fathers appear linked to moves over longer distances away from the ex-partner than for mothers. Utilising the behavioural and attitudinal detail held in the BHPS, we show that the distribution of pre-separation childcare responsibilities is relevant for determining post-separation proximity between parents: when both parents are jointly involved in pre-separation childcare, they maintain closer post-separation proximity. Finally, through the estimation of longitudinal trajectories of post-separation geographical proximity, our analysis suggests that the initial conditions around separation have long-lasting implications for the types of family life, ties and contact experienced in the years after separation. That is, most variation in the distance between ex-partners occurs in the immediate period following separation (approximately the first year), and thereafter, the distances tend to increase fairly modestly with time.

## Background

### Maintaining Existing Ties: Geographical Proximity of Separated Parents

By definition, the dissolution of a co-residential partnership will involve the relocation of at least one ex-partner from the joint home. A subsequent expectation could be that ex-partners sever their social and spatial ties and, as a result, gain relative independence in their post-separation mobility/migration careers. However, where shared children are involved, the simple expectation of post-separation independence is unlikely to hold—particularly in societies where shared parental custody and the involvement of fathers in childcare are commonplace (McGill 2014; Westphal et al. 2014). In the British context, separated parents are expected to make private childcare and residential arrangements, with only a small minority (≈10%) encouraged to seek mediation or council in order to agree such arrangements (Fehlberg et al. 2011). While it is common for children to spend a greater share of time with one parent (commonly referred to as the resident parent), estimates for the UK show 72% of non-resident parents self-report seeing their child at least several times a month (Fehlberg et al. 2011). In this context, desires to ensure regular child visitation, the sharing of parenting responsibilities and the well-being of shared children mean close geographical proximity between members of the post-separation family will tend to remain a critical concern (Stjernström and Strömgren 2012; Bakker and Mulder 2013; Viry 2014).

While rare, existing empirical analyses have revealed some evidence of continued spatial coordination between separated parents. For instance, drawing on large-scale population data for Sweden, Mulder and Malmberg (2011) found ex-partners with shared children to move significantly shorter distances from the former joint home than ex-partners without children. In Britain, Thomas et al. (2017) found separated parents to live in closer proximity than non-parental ex-partners in the approximate year following separation. And in the context of inter-state migration in the USA, Cooke et al. (2016) showed separated parents to have correlated migration propensities (i.e. where one remained/migrated, there existed a residual propensity for the other to do the same); no such correlation was found for ex-partners without children. While these rare examples are based on analyses of events and transitions at one time point only, they do emphasise the significance of shared children for encouraging geographical proximity in the post-dissolution family context.

Yet, despite the presence of shared children being shown to encourage constrained mobility and post-separation familial proximity, the specifics of post-separation child custody and residential arrangements are likely to encourage important variations within this overall pattern. Where both separated parents are actively involved in post-separation childcare, the maintenance of familial proximity is likely to be a strong and shared priority. However, where post-separation residential arrangements are spread more unequally between parents, the potential for the non-resident parent to relocate away from the former partner will presumably be increased. In some cases, gatekeeping practices by the resident parent (Dunn 2004)—preventing or restricting non-resident parents’ involvement—may undermine commitments to existing ties and encourage greater distances to emerge between the separated parents. Of course, commitments by non-resident parents need not be determined by gatekeepers, and some non-resident parents may simply be uninterested in the maintenance of ties and proximity. Yet, beyond these rather complex, diverse and difficult-to-measure issues, the simple presence of children in the home is known to constrain mobility, with desires to avoid upheavals to such things as child(ren)’s schooling and friendship networks being a major factor influencing parents’ (im)mobility decision-making (Green 1997; Fischer and Malmberg 2001; Bailey et al. 2004). Thus, where both separated parents retain resident children, moves away should be more restricted than in cases where only one parent bears primary resident-child responsibilities.

The degree to which both parents are actively involved in post-separation childcare responsibilities is likely to also be informed by the childcare dynamics prior to separation (Dunn 2004), though little empirical work currently exists on this topic. Where pre-separation parenting was shared, we would expect the desire and ability of both parents to maintain involvement, and thus proximity, to be increased. Indeed, where mothers continue to hold the primary caregiving responsibilities both pre- and post-separation, meta-analysis has shown that the pre-separation involvement of fathers with children is related to more frequent contact and better quality parent–child relationships after separation (Whiteside and Becker 2000). The sharing of pre-separation childcare should thus be related to closer proximity post-separation.

### Forging of New Family Ties

While the existing ties among members of the disbanded family can be thought a critical component of post-separation family mobility, the forging of new family ties is also likely to carry profound, and potentially competing, implications for residential (im)mobility decision-making and outcomes. Moves associated with repartnering represent a good example of a common dilemma facing separated parents. Of course, the formation of a new partnership may require an initial move into co-residence. Yet, beyond any initial move, the forming of a new household will also bring into the equation a new partner, and possibly his or her children, as additional decision makers with their own ties to different people and places. Such ‘blended families’—potentially stretching across multiple stepchildren, stepparents and locations—are a hallmark of contemporary family complexity (Sweeney 2010). While the importance of linked lives within complex families has remained largely unexplored within the empirical mobility/migration literature, the formation of new partnerships and the arrival of new post-separation children could be expected to compromise separated parents’ prioritisation of ties, commitments and proximity to the former household. Indeed, previous research suggests that the formation of new partnerships can have a negative impact on the negotiation of post-separation co-parenting and lead to reductions in frequency of visits with children (Anderson and Greene 2013). Beyond this, as time since separation increases, relations between non-resident family members are said to become less intimately linked (Dunn 2004), which would suggest that distances between separated parents should also increase with time.

### Gender Asymmetries

The concerns of family ties, geographical proximity and subsequent spatial constraints may fairly apply to separated parents regardless of their gender. Yet, in the family migration literature, taking a gendered perspective has long proved valuable (Bielby and Bielby 1992; Halfacree 1995; Cooke 2003, 2008). While it may be possible for any partner to be a ‘tied mover’ or ‘tied stayer’, women have tended to be overrepresented in sacrificing their own individual preferences in favour of the male partner. Previous research has shown how, for female partners to have an equal level of influence on family migration decisions, relative resources (e.g. human capital levels) often need to be stacked heavily in their favour (Cooke 2003; Compton and Pollak 2007). An important implication of this gender asymmetry is that, relative to men, women’s employment status, occupational careers and earnings tend to suffer after migration (e.g. Boyle et al. 2001; Clark and Huang 2006; Cooke et al. 2009)—though the negative effect of family migration on women’s careers may be short lived (Clark and Davies Withers 2002). While the aforementioned studies are focused on intact partnerships and families, the persistence of normative gender roles, the gendered expectations of care giving and the relative economic position of men and women in society permeate the post-separation family context too.

Despite the rise in shared parenting and the increased role of fathers in childcare (Fehlberg et al. 2011; McGill 2014; Westphal et al. 2014), mothers remain overwhelmingly more likely to hold the primary childcare and domestic responsibilities both before and after separation (Smerglia et al. 1999; Harris-Short 2011). In the UK, 91% of lone-parent households are headed by women (ONS 2015). The unevenness of these responsibilities can limit mothers’ opportunities in the waged labour market—traditionally a sphere of male dominance—and contribute to the generally poorer socio-economic position of women, relative to men (Jansen et al. 2009). They could also be expected to disproportionately limit separated mothers’ mobility careers, where the likelihood of being the primary caregiver means their (im)mobility decisions will tend to hold greater potential for impacting negatively on their children’s well-being (e.g. through the potential upheavals to child(ren)’s schooling and friendship networks mentioned above). It is also possible that normative gender expectations surrounding work and care responsibilities encourage separated fathers to be more open to moving away for career progression, repartnering and the formation of new families. Perhaps linked to this, separated men tend to repartner sooner and at higher rates than separated women (Dewilde 2008), with repartnering by non-resident fathers shown to reduce parent–child contact (Meggiolaro and Ongaro 2015)—N.B. interestingly, the latter study showed repartnering by non-resident mothers to be associated with greater non-resident parent–child contact. As such, we could expect that commitments to old family ties, and the desire or ability to take up new family ties, will vary between separated mothers and fathers. In general, we might expect the formation of new family ties by fathers to be associated with greater increases in the distance between the ex-partners than for mothers. Though again, where pre-separation childcare is more evenly split between mothers and fathers, the propensity for fathers to move away may be lessened.

### Geographical Contexts and Personal Resources

The decision-making and outcomes of spatial mobility are always embedded within broader macro-geographical structures (Mulder and Hooimeijer 1999). Here, the available stock and diversity in housing, repartnership, occupational and schooling options in the place of separation can be thought critical. More densely populated locations can be expected to offer more favourable options for separated parents to match their various locational needs, including the maintenance of close geographical proximity between them. In less densely populated areas, the stock and diversity of such factors will naturally be lower, with the ability to maintain proximity likely restricted (Thomas et al. 2017). Furthermore, in cases where both separated parents move out of the joint home at separation, finding two dwellings within close proximity that are suitable for children should prove more difficult and thus tend to lead to greater distances between the separated parents than where only one parent leaves the former joint home.

Beyond macro-geographical characteristics, personal resources and occupational factors can also be expected to frame the ability and/or desire to maintain familial proximity. The vast body of research into labour market migration demonstrates that levels of human capital attainment are important in influencing migration behaviour. Those with high attainment tend to migrate more frequently and over longer distances than those with lower attainment levels (Fielding 2012; Thomas et al. 2015; Stillwell and Thomas 2016). The classic explanation for this is that migration offers those with higher levels of human capital a generally greater potential return (in terms of career progression and earnings) than those with lower levels (Sjaastad 1962). Thus, in the context of post-separation family migration, the constraining nature of maintained familial proximity may be more clearly felt by those with higher attainment. It is possible that for separated parents with lower levels of attainment, the expected economic returns to migration will be less attractive and therefore less likely to compete with desires to maintain proximity. With regard to employment status and income levels, it is somewhat harder to think of a clear expectation. Indeed, those who are employed or have access to greater financial resources may be more likely to be able to afford to stay in the home or at least select accommodation that enables them to retain close familial proximity. With that said, it is also possible that the financial restrictions experienced by unemployed parents, or those with access to fewer resources, could also work to encourage proximity, though in this case via constraints as opposed to ‘choice-driven’ mechanisms.

### Data

For this longitudinal analysis, data are derived from Waves 1–18 (1991–2009) of the British Household Panel Survey (BHPS) with special licence access for lower-level geographical identifiers (ISER 2014).^1^ The survey was designed to collect data on a nationally representative sample of adult members (≈10,000) of households (≈5000) in Great Britain—N.B. Northern Ireland was not included until Wave 11 of the BHPS (Taylor et al. 2010) and is not included in our analysis. Longitudinal in design, the survey includes a broad range of questions on individual and household-level socio-economic and demographic characteristics and, in combination with detailed geographical identifiers, presents researchers with an opportunity to study the geographical distance between separated family members in the years following separation.

The analysis draws on an unbalanced panel sample of two-sex co-residential couples with children (dependent and non-dependent) that physically separate between any two waves and do not re-establish their partnership for the remaining period of data collection (hence couples are unbalanced in the number of post-separation waves they contribute). For inclusion in the sample, individual parents must have taken part in full interviews at wave *t* and be tracked to wave *t* + 1, where separated persons are identified and then matched to form ex-couple units. All waves are pooled (excluding Wave R which as the last wave of the BHPS does not allow a follow-up measurement at *t* + 1) which, after removing 6 ex-couple units due to missing geographical identifiers, produces an analytical sample of 402 parental ex-couples with 2477 wave occasions (Sample 1). In order to test the influence of pre-separation childcare responsibilities, a subsample (Sample 2) is drawn that includes an additional measure of the father’s perceived role in childcare prior to separation. This variable is not recorded in Wave C of the BHPS, which means the removal of 17 ex-couples (169 wave occasions). One ex-couple (9 wave occasions) is removed due to the fact that it is the only case where neither partner is recorded as performing the primary childcare responsibilities. Finally, item non-response on this variable also means that a further 70 ex-couples (402 wave occasions) are dropped. As such, the second analytical sample (Sample 2) contains 314 parental ex-couples with 1897 wave occasions.

While panel attrition and other forms of survey non-response are a recurring concern for survey-based analyses of separation and spatial mobility, the tracking procedures at the BHPS have proven successful in retaining a very high percentage of mobile respondents—e.g. where 15.1% of the sample required tracking from 2003 to 2004, the survey was successful in locating 93.7% of them (Couper and Ofstedal 2009). Among a range of tracking procedures, the most effective method is the use of details of yearly updated contact names who will know where the respondent is should they move (Laurie et al. 1999). To some extent, the very focus of our analysis is also likely to be of help. In the case of separation among families, we can expect a large share of separated parents to remain in contact, thus where one parent is recorded, locating the other should be simpler than in cases where no children exist. To affirm confidence in the sample, we performed comparisons of the characteristics of separating couples with children who remain in the sample against those who are lost to attrition. The results of this comparison (‘Appendix’) suggest that sample attrition is not highly selective and that our analyses should not be substantially biased by such issues. Previous checks on patterns of attrition related to separation and spatial mobility in the BHPS offer similar reassurance (e.g. Buck 2000; Uhrig 2008; Rabe and Taylor 2010; Fisher and Low 2012; Brewer and Nandi 2014). We include the strongest observed predictors of attrition in the analytical models (namely pre-separation socio-economic status, marital status and housing tenure).

The dependent variable in our analysis is the Euclidean distance (log km) between separated partners measured at each wave following separation. The distance is calculated using the centroids of the area of residence of each ex-partner, with Lower Super Output Areas (LSOA) used for England and Wales and Data Zones (DZ) for Scotland (Martin 2002). These equivalent small-area geographies contain an average of 1500 residents (650 households) and have an average area of 5.6 sq. km and radius of 0.76 km. In some cases, both ex-partners remain in the same geographical unit and so an estimate of the between-ex-couple distance is derived using the intra-zonal distance calculation of Batty (1976),^2^ which has been shown to be a reasonable approximation at detailed geographical scales (Stillwell and Thomas 2016).

The independent variables come in two forms: time varying and time constant. The time-constant variables, measured at the pre-separation wave, are used to characterise the joint household prior to separation. The time-constant variables, shown in Table 1, include: marital status (cohabiting or married); household employment configuration (working defined as being in a job; not working defined as unemployment, unpaid family care, retired or student status); housing tenure (social rent includes both local authority and housing association sectors; homeowner includes both outright owners and mortgage holders); household income (based on total household annual income); household (im)mobility at separation (whether the father, mother or both moved out at separation); and local area population density (defined as the logged population per hectare using Census 2001 aggregate data at the LSOA/DZ level). We also include a measure of household education configuration (based on the attainment of at least a bachelor’s degree-level education). Sensitivity analyses on different categorisations of educational attainment (e.g. also including O-level attainment) were performed, with the results suggesting that a degree-level education was the most important distinction for distances. This matches other studies performed in the British context, which show degree-level education to be consistently related to relocations over longer distances than lower-level qualifications (Boyle and Shen 1997; Fielding 2012; Thomas et al. 2015). An additional benefit of using a two-level educational variable (i.e. degree or no degree) is that it offers a parsimonious way of exploring any gender asymmetries pertaining to educational attainment (i.e. using: neither have a degree, both have a degree, only father has a degree, only mother has a degree). Beyond this, Sample 2 includes the additional measure of the father’s evaluation of who is responsible for pre-separation childcare (more the father, more the mother or jointly with partner). Finally, we may expect the age of the youngest shared child, or their status as a dependent child versus non-dependent child, to bear relevance to the proximity between separated parents. Our preliminary analyses revealed these factors to bear little substantive importance and so, for reasons of parsimony, they are not included in the analytical models below.

**Table 1:** Descriptive statistics of the two analytical samples

	Sample 1		Sample 2	
	Parental ex-couples: full		Parental ex-couples: subsample	
Time-constant variables	*n*_*j*_ = 402		*n*_*j*_ = 315	
Categorical	Freq.	%	Freq.	%
Household (im)mobility at separation				
Both move out	71	17.7	61	19.4
Father stays, mother moves out	116	28.9	84	26.7
Father moves out, mother stays	215	53.5	170	55.0
Marital status				
Married	289	71.9	215	68.3
Cohabiting	133	28.1	100	31.8
Household employment configuration before separation				
Both working	233	58.0	169	53.7
Neither working	45	11.2	39	12.4
Father not working, mother working	33	8.2	29	9.2
Father working, mother not working	91	22.6	78	24.8
Household education configuration before separation				
Both have degree	14	3.5	7	2.2
Father degree, mother no degree	27	6.7	20	6.4
Father no degree, mother degree	18	4.5	17	5.4
Neither have degree	343	85.3	271	86.0
Tenure of home before separation				
Homeowner	251	62.4	188	59.7
Private rent	42	10.5	37	11.8
Social rent	109	27.1	90	28.6
Household income before separation (percentile)				
Below 25th	79	19.7	66	21.0
25th–49th	121	30.1	102	32.4
50th–74th	111	27.6	90	28.6
75th and above	91	22.6	57	18.1
Fathers perceived childcare involvement before separation				
Neither partner (someone else)^a^			1	0.3
Joint with partner			119	37.8
Father more			17	5.4
Mother more			178	56.5

Continuous	Mean	SD	Mean	SD
Population density (log population per hectare)	2.7	1.5	2.8	1.5

Time-varying variables	*n*_*ij*_ = 2477		*n*_*ij*_ = 1906	
Categorical	Freq.	%	Freq.	%
Post-separation new partnership configuration				
Both single	1149	46.4	868	45.5
Both new partners	523	21.1	392	20.6
Father new partner, mother single	450	18.2	387	20.3
Father single, mother new partner	355	14.3	259	13.6
New post-separation child(ren) configuration				
Neither new child	1673	67.5	1227	64.4
Both new child	168	6.8	159	8.3
Father new child, mother no new child	390	15.7	292	15.3
Father no new child, mother new child	246	9.9	228	12.0
Residence of pre-separation child(ren) configuration				
Child(ren) no longer with parents	194	7.8	42	2.2
Both have child(ren)	275	11.1	221	11.6
Father has child(ren), mother no child(ren)	179	7.2	122	6.4
Father no child(ren), mother has child(ren)	1829	73.8	1521	79.8

Continuous	Mean	SD	Mean	SD
Time since separation (approximate to years)	4.2	3.7	4.1	3.6
Distance separating ex-partners				
Log kilometres (dependent variable)	1.5	1.9	1.5	1.9
Kilometres	25.9	65.4	28.1	70.6
Distance separating ex-partners (kilometres) by time				
Year == 0 (initial distance upon separation)	1.3	1.7	1.3	1.7
Year == 2	1.4	1.8	1.3	1.9
Year == 4	1.6	1.9	1.6	1.9
Year == 6	1.7	1.8	1.8	1.9

N.B. percentages may not sum to 100 due to rounding

^a^ Indicates the 1 ex-couple (and 9 repeated observations) removed in sample 2

The major benefit of utilising multiple waves of data is that we can include a series of time-varying variables designed to establish how the formation of new family ties may interact with the maintenance of existing ones. Described in Table 1, the three time-varying variables are designed to record new post-separation partnerships (both new partners; father new partner and mother single; mother new partner and father single; and both single); new post-separation child(ren) (both new child(ren); father new child(ren), mother no new child(ren); father no new child(ren), mother new child(ren); neither new child(ren)); and as a measure of child custody/residency arrangements, the recorded residence of pre-separation child(ren) (both have child(ren); father has child(ren), mother has no child(ren); father has no child(ren), mother has child(ren); child(ren) no longer with parents. Finally, in order to enable the estimation of longitudinal trajectories of post-separation geographical proximity, we include a measure of time since separation (based on post-separation wave occasions—approximate to years) and time since separation squared (to allow for nonlinear trajectories).

### Method

The analysis draws on random-effects models with random intercepts, random slopes (coefficients) and a first-order autoregressive structure for residual dependence (Snijders and Bosker 2012). When applied to panel data, random-effects models, often called growth curve models, provide the ability to identify developmental trajectories (e.g. in the distance between separated parents) over some measure of time.

In the case of the two-level models below, where repeated wave occasions (level 1) are nested within ex-couple units (level 2), the random-effects approach offers important analytical advantages over the more commonly used fixed-effects panel approach. For instance, the (level-1) coefficient for linear time can be allowed to vary between (level-2) ex-couples, thus enabling a measurement of heterogeneity in the ex-couple distance trajectories (the slopes) and their baselines (the intercepts). Also, unlike fixed-effects models, time-constant ex-couple characteristics (i.e. all pre-separation characteristics) can be easily incorporated. Random-effects models have tended to be avoided due to problems of endogeneity between the time-varying coefficients and the time-invariant residual term. However, as demonstrated by Bell and Jones (2015), a variant of the Mundlak formulation, breaking up time-varying variables into *within effects* and *between effects*, can be used to avoid such issues. The *within effect* is calculated as a group-mean-centred covariate $(x_{ij}-{{\bar{x}}_{j}})$ and has an interpretation equivalent to a fixed-effects model estimate. Importantly, the within effect provides a more robust estimate of within ex-couple change, having accounted for the observed time-varying covariates as well as observed and unobserved (residual) time-constant characteristics. The *between effect* is calculated as the group-mean of the covariate ($\bar{x}_{j}$), and for categorical variables is calculated as the proportion of time spent in a given category over the period of observation. Between effects can offer substantive value in some empirical examples (see Bell and Jones 2015)—e.g. giving the average effect of being member of a given category for all ex-couples across all waves.^3^

A simplified form of the random-effects model employed in the analysis is presented in Eq. 1, with a single time-varying variable (e.g. time since separation) divided into constituent within and between elements, a single time-constant variable (e.g. father’s perceived childcare involvement prior to separation), random intercepts, random slopes and autocorrelated residuals:

1 $$\begin{aligned} y_{ij} &= \beta_{0} + \beta_{1j} \left( {x_{ij} - \bar{x}_{j} } \right) + \beta_{2} \bar{x}_{j} + \beta_{3} x_{j} + \left[ {u_{0j} + u_{1j} \left( {x_{ij} - \bar{x}_{j} } \right) + e_{ij} } \right] \hfill \\ \left[ {\begin{array}{*{20}c} {u_{0j} } \\ {u_{1j} } \\ \end{array} } \right] &\sim N\left( {0,\left[ {\begin{array}{*{20}c} {\sigma_{u0}^{2} } & {} \\ {\sigma_{u01} } & {\sigma_{u1}^{2} } \\ \end{array} } \right]} \right) \hfill \\ e_{ij} &\sim N\left( {0,{\varvec{\Omega}}_{e} } \right) \hfill \\ \end{aligned}$$

where *y*_*ij*_ is the distance (log km) between ex-couple *j* at wave occasion *i*, *β*_0_ is the overall intercept and represents the average distance across all *i* and *j* units when all variables are held at their reference value. *β*_1*j*_ (measured at level 1) is the estimated average within-effect slope term associated with the time-varying variable (i.e. linear time). Here the *j* subscript denotes that this coefficient is allowed to vary across all level-2 units, in this case enabling each ex-couple to have their own time-dependent distance trajectory. *β*_2_ (measured at level 2) is the estimated average between - effect ($\bar{x}_{j}$) slope term for the same predictor variable. *β*_3_ (measured at level 2) is the estimated slope term for a time-constant predictor variable (*x*_*j*_). Meanwhile, *u*_0*j*_ and *u*_1*j*_ represent the conditional random differential intercepts term and random coefficient term. These level-2 random effects are assumed to follow a bivariate normal distribution with a zero mean, variances *σ*_*u*0_^2^ and *σ*_*u*1_^2^ and a covariance *σ*_*u*01_, which reflects the covariation between the random intercepts and slopes. With autocorrelated residuals, *e*_*ij*_ is assumed to follow a normal distribution with zero mean and a residual covariance matrix for the repeated wave occasions (${\varvec{\Omega}}_{e}$)—containing equal variance (*σ*_*e*_^2^) and off diagonal covariances that are the product of the variance and the autocorrelation coefficient (*ρ*) raised to increasing powers as wave occasions become increasingly separated by time and therefore less dependent (Jones and Subramanian 2013).^4^ The autocorrelation coefficient *ρ* gives the correlation between consecutive wave occasions *i* and *i*′ ($corr\left( {e_{ij} ,e_{{i^{\prime}j}} } \right) = \rho^{{\left| {i - i^{\prime}} \right|}}$) and is assumed to be constant for a given time lag (Steele 2014). Alternative specifications of the residual covariance matrix were tested with the first-order autoregressive structure found to be the most parsimonious and appropriate for this analysis.

## Results

Table 2 presents the results of the initial analysis of variations in the distance between separated parents using Sample 1. As expected, the within effect of time suggests that the distance between ex-partners with shared children increases with each year (*exp*(0.093) = 1.098 = 9.8%). The quadratic term (time-squared) indicates that the rate of increase declines very slightly with time, though the estimated 95% confidence interval for this estimate includes zero. With the time coefficients being group-mean centred and therefore referring to change from the middle observation of each ex-couple, the estimated km change from the baseline point, if we include both linear and quadratic terms, can be calculated as: exp (1.983 + 0.093 + 2* − 0.003) − exp (1.983) = 0.66 km. The random coefficient for linear time in Table 2 indicates that the distance trajectories are rather consistent between ex-couples, with a relatively small amount of variation observed in the slope terms (*σ*_*u*1_^2^). Indeed, the majority of residual variation is found in the random intercepts (*σ*_*u*0_^2^): the conditional 95% coverage interval suggests that ex-partners at the 97.5th percentile of the intercept distribution have an estimated distance of approximately 85 km between them, whereas ex-partners at the 2.5th percentile of this distribution have an estimated proximity of just 0.62 km.^5^ There is some suggestion of a positive covariance (*σ*_*u*01_) between the slopes and intercepts indicating that those with greater initial distances are more likely to have greater growth trajectories too—though again, the size of the random slope and covariance coefficients are small and therefore of little substantive importance. Acknowledging the trivial variation in the slope terms, the initial distance (measured at the intercept) appears to provide a very good indication of the subsequent proximities families will maintain in the period that follows. As such, the initial conditions around separation appear to have long-lasting implications for the types of family life, ties and contact subsequently experienced.

**Table 2:** Distance (log km) separating parental ex-partners following separation

	Coef.	Std. Err.	Lower 95% CI	Upper 95% CI
Constant	1.983*	0.256	1.482	2.484
Time since separation (wi)	0.093*	0.025	0.044	0.143
Time since separation squared (wi)	−0.003	0.002	−0.007	0.001
Marital status (ref: *Married*)				
Cohabiting	−0.008	0.170	−0.340	0.324
Household employment before separation (ref: *Both working*)				
Neither working	0.079	0.292	−0.493	0.651
Father not working, mother working	0.140	0.278	−0.404	0.684
Father working, mother not working	0.179	0.189	−0.192	0.550
Household education before separation (ref: *Neither have degree*)				
Both have degree	1.014*	0.407	0.215	1.812
Father has degree, mother has no degree	0.500	0.297	−0.083	1.083
Father has no degree, mother has degree	−0.366	0.359	−1.070	0.338
Tenure of home before separation (ref: *Homeowner*)				
Private rent	0.042	0.257	−0.462	0.545
Social rent	−0.246	0.204	−0.646	0.155
Household income before separation (ref: *25th*–*49th percentile*)				
Below 25th	−0.143	0.227	−0.588	0.303
50th–74th	−0.155	0.197	−0.540	0.230
75th and above	−0.141	0.219	−0.570	0.288
Household (im)mobility at separation (ref: *Father moves out, mother stays*)				
Both move out	0.709*	0.201	0.315	1.103
Father stays, mother moves out	−0.035	0.171	−0.371	0.300
Population density (log population per hectare)	−0.286*	0.048	−0.381	−0.192
Post-separation new partnership (ref: *Both single*)				
Both new partners (wi)	−0.358*	0.084	−0.522	−0.195
Father new partner, mother single (wi)	−0.158	0.081	−0.316	0.001
Father single, mother new partner (wi)	−0.019	0.076	−0.169	0.131
Both new partner (bw)	0.224	0.265	−0.295	0.742
Father new partner, mother single (bw)	0.879*	0.268	0.354	1.404
Father single, mother new partner (bw)	0.380	0.297	−0.202	0.962
New post-separation child(ren) (ref: *Neither new child*)				
Both new child(ren) (wi)	−0.014	0.139	−0.258	0.287
Father new child(ren), mother no new child(ren) (wi)	0.527*	0.095	0.341	0.713
Father no new child(ren), mother new child(ren) (wi)	0.172	0.108	−0.040	0.385
Residence of pre-separation child(ren) (ref: *Father no child(ren), mother has child(ren)*)				
Child(ren) no longer with parents (wi)	−0.201	0.138	−0.472	−0.070
Both have child(ren) (wi)	−1.014*	0.094	−1.198	−0.829
Father has child(ren), mother has no child(ren) (wi)	−0.322*	0.126	−0.569	−0.076
Level-2 random-effects parameters				
*σ*_*u*0_^2^ (Intercept variance)	1.584	0.188	1.255	2.000
*σ*_*u*1_^2^ (Time (wi) slope variance)	0.018	0.005	0.010	0.032
*σ*_*u*01_ (Intercept–time (wi) covariance)	0.090	0.021	0.049	0.131
Level-1 residual: AR(1)				
*ρ*	0.651	0.044	0.557	0.729
Variance (Residual)	1.061	0.136	0.826	1.364
Log likelihood	−3357.0525			
Wald *χ* ^2^ (degrees of freedom)	315.46 (29)			

N.B. *wi* = within effect; bw = between effect. Sample 2: level-2 $n_{j}$ = 402, level-1 *n*_*ij*_ = 2477. The large residual autocorrelation (*ρ* = 0.651) suggests that the AR(1) residual structure is necessary for accounting for intra-ex-couple dependency

* Indicates fixed-part estimates are statistically significant at the 95 percent level

With regard to the time-constant variables, we see that household (im)mobility at separation is particularly important. As expected, when both parents leave the former joint home the distance between them is greater than when only one leaves (*e* ^0.709^ = 2.0 times greater than the reference category, where the father moves out and mother stays). Where we assume the majority of parents desire to maintain close proximity, this finding is presumably linked to the relative difficulties associated with the locating of two new and suitable dwellings within close proximity, as opposed to just one. Similarly, where macro-geographical opportunity structures can be thought more favourable in more densely populated areas, we find shorter distances are associated with separations that occur in areas of greater population density.

There is little evidence of any substantively important variations according to marital status, household employment status or household income. However, we do observe the expected positive relationship between high human capital attainment and increased distances between parents. There is also some hint that separated fathers with higher educational attainment may be more likely to move away than otherwise similar mothers, though the distribution of this variable means that these estimates are based on few cases and are thus accompanied by particularly large standard errors.

The time-varying variables provide us the opportunity to study the balance between maintaining exiting family ties and forging new ones, as well as any variations that may exist between mothers and fathers therein. Starting with the post-separation residence of shared (pre-separation) children, we see that the distance between separated parents is almost 3 times shorter (*e* ^1.014^ = 2.8) when both have a child(ren) resident as compared to when only the mother has the shared child(ren). With the single largest effect size, this finding fits with the argument that resident children constrain mobility. Moreover, as a rough measure for shared custody arrangements, it also fits with the notion that maintaining shared parental involvement reinforces the willingness of parents to remain in close proximity and coordinate their (im)mobility careers. Indeed, where only one parent has the child(ren), the desire and/or ability of the non-resident parent to relocate appears increased. Interestingly, we find that in cases where only the father retains children in the home, the distance between parents is shorter than where only the mother has the children. Thus, there is some suggestion that non-resident mothers are less willing or able to compromise on proximity than non-resident fathers.

Gender asymmetries are also present in the formation of new family ties. When the father has a new (post-separation) child, the distance to the former partner is significantly increased (*e* ^0.527^ = 1.7 times). This finding matches previous research showing that the arrival of children with a new partner is associated with reductions in fathers’ contact with children from their previous partnerships (Manning and Smock 1999). In terms of the formation of new co-residential partnerships, the within-effect estimates suggest that a transition from being single into a new partnership is associated with closer proximity. While the direction of the within-effect relationship is difficult to explain, the between-effect estimates for the post-separation partnership configuration do fit with our expectations—N.B. preliminary analyses showed the between effects for the other time-varying variables to be in the same direction as their within effects; offering little substantive interest, we exclude them to reduce model complexity. Interpreted as average effects, the between effects suggest that distances are greater when separated parents spend a greater proportion of the post-separation period repartnered, as opposed to single. Again, a particularly large effect is found when the father spends more of the post-separation period repartnered. Taken together, it would appear that the formation of new family ties does have implications for the maintenance of old family ties, though it also appears that fathers are the more willing and/or able to compromise on existing ties, commitments and proximity.

Table 3 shows the results of an analysis that includes the father’s perceived childcare responsibilities at the wave prior to separation. While the overall substantive findings remain the same, the inclusion of this variable fits with our prior expectations. That is, where the father perceived that childcare was performed jointly, the distance is found to be significantly shorter (*e* ^0.374^ = 1.5 times) than when the mother was the main provider of care. This finding provides us with a rare empirical demonstration of the relevance of pre-separation childcare dynamics for post-separation family ties and proximity.

**Table 3:** Distance (log km) separating parental ex-partners following separation (including fathers perceived pre-separation childcare involvement)

	Coef.	Std. Err.	Lower 95% CI	Upper 95% CI
Constant	2.012*	0.300	1.424	2.601
Time (wave-years) (wi)	0.105*	0.030	0.047	0.163
Time^2^ (wi)	−0.003	0.003	−0.008	0.002
Fathers perceived childcare involvement prior to separation (ref: mother more)				
Joint with partner	−0.374*	0.182	−0.730	−0.018
Father more	0.502	0.392	−0.265	1.271
Marital status (ref: *Married*)				
Cohabiting	0.001	0.190	−0.371	0.374
Household employment before separation (ref: *both working*)				
Neither working	0.249	0.338	−0.414	0.912
Father not working, mother working	0.008	0.331	−0.641	0.656
Father working, mother not working	0.084	0.222	−0.351	0.519
Household education before separation (ref: *neither have degree*)				
Both have degree	1.245*	0.559	0.148	2.341
Father has degree, mother has no degree	0.527	0.353	-0.164	1.219
Father has no degree, mother has degree	−0.495	0.391	−1.261	0.272
Tenure of home before separation (ref: *homeowner*)				
Private rent	0.101	0.291	−0.469	0.670
Social rent	−0.262	0.236	−0.723	0.200
Household income before separation (ref: *25th to 49th percentile*)				
Below 25th	−0.314	0.258	−0.819	0.192
50th to 74th	−0.097	0.224	−0.535	0.341
75th and above	−0.072	0.270	−0.602	0.457
Household (im)mobility at separation (ref: *father moves out, mother stays*)				
Both move out	0.799*	0.225	0.359	1.239
Father stays, mother moves out	0.009	0.201	−0.385	0.403
Population density (*log population per hectare*)	−0.259*	0.057	−0.369	−0.148
Post-separation new partnership (ref: *both single*)				
Both new partners (wi)	−0.362*	0.097	−0.553	−0.171
Father new partner, mother single (wi)	−0.179*	0.091	−0.357	−0.001
Father single, mother new partner (wi)	−0.186*	0.089	−0.360	−0.011
Both new partner (bw)	0.160	0.308	−0.443	0.764
Father new partner, mother single (bw)	0.764*	0.299	0.179	1.349
Father single, mother new partner (bw)	0.437	0.367	−0.282	1.155
New post-separation child(ren) (ref: *neither new child*)				
Both new child(ren) (wi)	−0.029	0.148	−0.319	0.261
Father new child(ren), mother no new child(ren) (wi)	0.434*	0.107	0.223	0.644
Father no new child(ren), mother new child(ren) (wi)	0.183	0.120	−0.053	0.419
Residence of pre-separation child(ren) (ref: *father no child(ren), mother has child(ren)*)				
Child(ren) no longer with parents *(wi)*	−0.167	0.248	−0.652	0.319
Both have child(ren) (wi)	−1.168*	0.108	−1.379	−0.957
Father has child(ren), mother has no child(ren) (wi)	−0.585*	0.165	−0.908	−0.261
Level-2 random-effects parameters				
*σ*_*u*0_^2^ (Intercept variance)	1.607	0.215	1.237	2.087
*σ*_*u*1_^2^ (Time (wi) slope variance)	0.016	0.006	0.008	0.034
*σ*_*u*01_ (Intercept–time (wi) covariance)	0.084	0.024	0.037	0.130
Level-1 residual: AR(1)				
*ρ*	0.652	0.049	0.545	0.738
Variance (residual)	1.087	0.157	0.820	1.441
Log likelihood	−2590.0173			
Wald *χ* ^2^ (degrees of freedom)	292.74 (31)			

N.B. wi = within effect; bw = between effect. Sample 2: level-2 *n*_*j*_ = 314, level-1 *n*_*ij*_ = 1897. The large residual autocorrelation (*ρ* = 0.652) suggests that the AR(1) residual structure is necessary for accounting for intra-ex-couple dependency

* Indicates fixed-part estimates are statistically significant at the 95 percent level

## Conclusion

While existing literature has proven valuable in demonstrating the spatially constrained nature of post-separation family mobility, much of this work has drawn on short snapshots of data, offering little potential for the analysis of longer-term mobility careers of separated families. In breaking with this tradition, this paper combines 18 years of BHPS data with longitudinal random-effects models in order to define and test the persistence and significance of family ties, both new and old, for post-separation (im)mobility. An important finding of our analysis relates to the way in which post-separation linked lives, proximities and spatial constraints are characterised by important gender asymmetries. Indeed, the formation of new family ties (partners and children) by fathers is found to be linked to moves over longer distances away from the ex-partner than is the case for mothers. Where the family migration literature has highlighted the overrepresentation of women as tied spouses (sacrificing their individual (im)mobility preferences in favour of the male partner), it would appear that mothers are also particularly constrained in the post-separation context. Where spatial mobility provides a means through which people can match their location to their broader needs, the increased constraints experienced by mothers may be important in limiting opportunities for post-separation adjustment, recovery and well-being. Indeed, this could be an important, and currently underappreciated, factor behind the socio-economic inequalities observed between men and women in the post-separation context.

Our analysis also suggests that the distribution of pre-separation childcare responsibilities, as described by the father, is relevant for determining post-separation proximity between parents. When both parents were reported as jointly involved in childcare prior to separation, the ex-partners tend to live in closer proximity post-separation. Moreover, from the perspective of our time-varying measure of the post-separation residence of shared (pre-separation) children, closer proximity is also observed when both parents have their child(ren) resident. The very presence of children is known to constrain mobility, yet as a rough measure for shared custody arrangements, our findings suggest that maintaining shared parental involvement reinforces the willingness of separated parents to coordinate their residential locations and remain in close proximity. In cases where only one parent has the shared child(ren) present, the distance between parents is found to be shorter when the non-resident parent is the mother. This again fits the gendered theme of our findings, with non-resident mothers seemingly less willing or able to compromise on proximity than non-resident fathers.

More broadly, the estimation of longitudinal trajectories of post-separation geographical proximity indicates that the initial conditions and outcomes around separation have long-lasting implications for the types of family life, ties and contact experienced in the years after separation. We find most variation in the distance between ex-partners to occur within a period approximate to a year after separation, with the degree of proximity varying only modestly between ex-couples with time. While there is a general trend for distances to increase with time, it is clear that those who initially move far apart tend to remain far apart, while those who move only short distances apart tend to maintain their proximity.

For future research, it could be particularly useful to undertake similar studies in different national contexts: with differing welfare regimes, gender expectations, male and female labour market positions and national/regional housing markets. It may be the case that certain national contexts encourage greater or reduced proximities, as well as more or less gendered outcomes, than we observe for Britain. Even within advanced Western economies, important differences can be expected when comparing more conservative nations, such as Germany, to the more social-democratic ones, for instance those of Denmark, Norway and Sweden. Moreover, while often less detailed in terms of the individual/household attributes covered, geocoded population data could be useful for testing the generalisability of some of the findings of this analysis. A second avenue for future discussion and research could be to identify and understand the role that greater extended-family complexity and spatial ties can have on macro-migration processes and dynamics. While the microrelationships appear to show clear evidence of spatial constraints on separated parents, the contemporary ubiquity of family instability and complexity could be an important factor in shaping current and future patterns of migration and population (re)distribution. Indeed, the spatial constraints associated with more complex family ties mean researchers and policymakers should be aware of potential future reductions in the ability of migration and mobility to act as efficient allocators of individuals within regional labour and housing markets.

## Appendix

The table below shows the characteristics of a GB (excluding Northern Ireland) sample of co-residential two-sex couples who separate between *t* (wave prior to separation) and *t* + 1 (wave after separation), comparing those who are tracked to those who attrit. Separations are identified using the household grid file of the BHPS, with partners who live in different households at wave *t* + 1, and who do not get back together in the subsequent waves, selected. Where both partners are lost to follow-up, it is not possible to know whether they separated. N.B. separations do not include cases where one of the partners dies. All waves (1991–2009) are pooled (N.B.—it is not possible to include separations that may occur after Wave *R* as this is the last wave of the BHPS and so does not allow a measurement at t + 1).

In the complete-case samples (CC) below, all respondents must complete full interviews at *t*, the separating couples with children form the basis of our analytical sample. The raw samples are used for comparison, containing all recorded ex-couples and reporting the basic socio-demographic information that can be obtained without full interview participation (thus including proxy responses and refusals that are removed in CC analysis). Note that in the raw samples, the variables housing tenure and household employment configuration do contain some missing cases.

Unweighted descriptives for co-residential couples separating between t and t + 1 by survey follow-up status (tracked/attrit).

**Table:**

Variables (measured at *t*)	All separating couples (raw)		Separating couples with child(ren) (raw)		All separating couples (CC)		Separating couples with child(ren) (CC)	
	Tracked (*n* = 576)	Attrit (*n* = 471)	Tracked (*n* = 448)	Attrit (*n* = 360)	Tracked (*n* = 524)	Attrit (*n* = 293)	Tracked (*n* = 408)	Attrit (*n* = 226)
Age (couple-mean average years)	35.9	35.9	34.9	33.7	35.5	34.3	34.9	32.6
Marital status (%)								
Cohabiting	31.6	38.9	28.4	38.9	31.9	38.6	27.9	36.7
Black and minority ethnic (%)					5.1^5^	4.7^6^	3.6^7^	6.1^8^
Household type (%)								
Couple-dependent child	72.2	72.2			72.7	73.7		
Couple-non-dependent child	5.6	4.3			5.2	3.4		
Couple: no child	21.5	22.3			21.4	22.5		
Other household	0.7	1.3			0.0	0.0		
Interview year (%)								
1991–1996	36.6	28.5	31.5	27.2	37.2	30.0	31.4	28.8
1997–2002	37.9	44.6	39.7	43.6	38.2	44.0	40.4	42.5
2003–2007	35.5	26.9	28.2	29.2	24.6	25.9	28.2	28.8
Region *t* (%)								
East Midlands	9.0	8.1	9.6	8.3	9.2	9.2	9.8	10.6
East of England	8.7	5.3	9.2	5.8	9.0	4.4	9.3	4.9
London	5.4	4.9	4.5	4.7	5.2	3.8	4.2	4.0
North-east	5.2	4.0	4.2	4.4	5.2	4.8	4.2	5.8
North-west	10.6	8.5	10.3	8.9	10.9	7.9	10.8	6.6
Scotland	13.7	17.4	13.0	15.6	13.9	16.4	12.8	13.7
South-east	12.3	7.6	11.4	8.1	11.6	8.2	10.5	8.4
South-west	6.7	6.0	8.3	5.8	6.7	6.8	8.3	7.1
Wales	14.1	19.8	15.6	18.6	13.7	19.8	15.2	18.6
West Midlands	6.3	9.6	6.3	11.1	6.5	9.9	6.6	11.5
Yorkshire and the Humber	7.8	8.9	7.8	8.6	8.4	8.9	8.3	8.8
Household education (%)								
Degree					16.2	10.9	14.5	9.3
Household employment (%)								
Both working	59.1^1^	52.7^2^	57.5^3^	51.1^4^	60.3	53.2	58.3	49.6
Only woman working	7.8^1^	6.9^2^	8.1^3^	6.7^4^	8.0	7.9	8.1	7.5
Only man working	20.2^1^	21.9^2^	23.7^3^	25.3^4^	19.5	20.1	22.6	24.8
Neither working	12.9^1^	18.5^2^	10.7^3^	16.9^4^	12.2	18.8	11.0	18.1
Sample membership								
Original sample	79.9	70.8	79.9	73.6	79.8	73.0	79.7	76.1
Echp—ons	1.4	1.1	1.6	1.1	1.5	1.4	1.7	1.8
Echp—scpr	1.2	3.0	0.9	3.1	1.0	4.1	1.0	4.0
Scotland new sample	8.0	9.8	7.1	8.6	8.2	7.2	7.4	5.8
Wales new sample	9.6	15.5	10.5	13.6	9.5	14.3	10.3	12.4
Household income (%)								
Below 25th percentile	21.2	31.2	19.9	32.8	20.4	29.7	19.6	32.3
25th to 50th percentile	27.6	26.5	29.7	26.4	27.9	28.7	30.1	28.8
50th to 75th percentile	26.0	21.9	27.2	21.7	26.7	21.8	27.7	20.8
Above 75th percentile	25.2	20.4	23.2	19.2	25.0	19.8	22.6	18.1
Housing tenure (%)								
Homeownership	65.9^1^	59.1^2^	70.0^3^	56.7^4^	65.5	57.4	62.8	54.4
Social rent	23.7^1^	32.0^2^	28.8^3^	35.1^4^	23.9	32.8	27.0	9.3
Private rent	10.5^1^	8.8^2^	11.1^3^	8.2^4^	10.7	9.9	10.3	36.3
Distance moved by female movers (median km)	3.5	4.8	2.6	3.5	3.7	4.6	3.0	2.8
Mobility rate (*t* + 1) (%)								
Male ex-partner	66.2		69.9		66.4		70.6	
Female ex-partner	51.6		47.1		51.3		46.3	
Attrition by gender (%)								
Male ex-partner lost to follow-up		80.3		84.2		78.5		81.9
Female ex-partner lost to follow-up		29.7		25.8		32.8		28.8

N.B. Echp—ons and Echp—scpr represent the European Community Household Panel subsample that was integrated into the BHPS in 1997 and discontinued in 2001. The Scotland and Wales new samples are booster samples introduced in 1999. The distance moved by male movers is not included in the table above as there are only 8 couples where the mother attrits and the father moves and remains in the sample

^1^ Indicates the denominator is 574 due to 2 missing cases, ^2^ indicates the denominator is 465 due to 6 missing cases, ^3^ indicates the denominator is 356 due to 4 missing cases, and ^4^ indicates the denominator is 447 due to 1 missing case. Note that, while not used in the analysis, the CC samples contain item non-response on the Black and minority ethnic category (^5^ indicates the denominator is 524 due to 18 missing cases, ^6^ indicates the denominator is 279 due to 14 missing cases, ^7^ indicates the denominator is 391 due to 17 missing cases, and ^8^ indicates the denominator is 212 due to 14 missing cases

Comparing between the raw and CC samples, and within the samples between those who attrit and those who remain, results prove reassuring. While attrition rates are quite high in total, and while men tend to be lost to attrition more than women, there is good comparability between households who remain in the sample and those who are lost to attrition. Indeed, evidence of household-level selectivity appears to be limited to pre-separation socio-economic status (income and employment status), marital status and housing tenure. This fits with the many previous checks on attrition associated with separation and spatial mobility (see Buck 2000; Uhrig 2008; Rabe and Taylor 2010; Fisher and Low 2012; Brewer and Nandi 2014). Nevertheless, we include these predictors of attrition in our analytical models.

The final analytical sample (Tables 1, 2) contains 402 ex-couples as a result missing geographical identifiers (LSOA) at *t* + 1, which prevents the calculation of between ex-couple distance.

## Footnotes

1 While the BHPS sample was subsumed into Wave 2 of Understanding Society (UKHLS), issues of high rates of attrition (associated with geographical mobility and transition into the new survey) and differing survey design means, we limit our analysis to the original BHPS waves only.

2 Where the between distance in zone *i* is calculated as $r_{i} /\sqrt 2$, where *r* is the radius of a circle equivalent in area (*A*) to zone *i* and where the radius can be defined as $\sqrt {A/\pi }$ (Stillwell and Thomas 2016).

3 However, with few repeated measures and the potential for unobserved confounding, substantive interpretations of between effects should be made with care. See Longford (1989) for a discussion on the issues of quality of group-mean coefficients.

4 For repeated measures data with 5 wave occasions:

$${\varvec{\Omega}}_{e} = \left( {\begin{array}{*{20}c} {\sigma^{2} } & {} & {} & {} & {} \\ {\sigma^{2} \rho } & {\sigma^{2} } & {} & {} & {} \\ {\sigma^{2} \rho^{2} } & {\sigma^{2} \rho } & {\sigma^{2} } & {} & {} \\ {\sigma^{2} \rho^{3} } & {\sigma^{2} \rho^{2} } & {\sigma^{2} \rho } & {\sigma^{2} } & {} \\ {\sigma^{2} \rho^{4} } & {\sigma^{2} \rho^{3} } & {\sigma^{2} \rho^{2} } & {\sigma^{2} \rho } & {\sigma^{2} } \\ \end{array} } \right),$$

which is equivalent to a correlation structure:

$$\left( {\begin{array}{*{20}c} 1 & {} & {} & {} & {} \\ \rho & 1 & {} & {} & {} \\ {\rho^{2} } & \rho & 1 & {} & {} \\ {\rho^{3} } & {\rho^{2} } & \rho & 1 & {} \\ {\rho^{4} } & {\rho^{3} } & {\rho^{2} } & \rho & 1 \\ \end{array} } \right)$$

5 Conditional 95% coverage interval for intercepts calculated as: $\left( { - 1.96\sqrt {\sigma_{u0} } , + 1.96\sqrt {\sigma_{u0} } } \right) = \left( { - 1.96\sqrt {1.584} , + 1.96\sqrt {1.584} } \right) = \left( { - 2.47, + 2.47} \right)$ (N.B. values here rounded to 2 decimal places). These values can then be added to the overall intercept value and exponentiated to get the 2.5th and 97.5th percentile estimates in km terms.
